# (*Z*)-*N*-{3-[(2-Chloro-1,3-thia­zol-5-yl)meth­yl]-1,3-thia­zolidin-2-yl­idene}cyanamide

**DOI:** 10.1107/S1600536811047404

**Published:** 2011-11-12

**Authors:** Yue-Ming Li, Jian-Ye Li, Qian Wang, Ai-You Hao, Tao Sun

**Affiliations:** a School of Chemistry and Chemical Engineering, Shandong University, Jinan 250100, People’s Republic of China; b Chemical Engineering Department, Weifang University of Science and Technology, Shouguang 262700, People’s Republic of China; c Beijing University of Chemical Technology, Beijing 100029, People’s Republic of China

## Abstract

In the title compound, C_8_H_7_ClN_4_S_2_, the thia­zole ring is essentially planar [r.m.s. deviation = 0.0011 (2) Å] and conformation of the thia­zolidine ring is twisted on the C—C bond. The C=N bond has a *Z* configuration.

## Related literature

The title compound was synthesized as an inter­mediate for the preparation of pesticides. For the biological activity of this class of compounds, see: Zhang *et al.* (2000 ▶); Kagabu *et al.* (2008 ▶). For the synthesis, see: Kozo *et al.* (1987 ▶); Zuo *et al.* (2008 ▶). For a related structure. see Li *et al.* (2010 ▶).
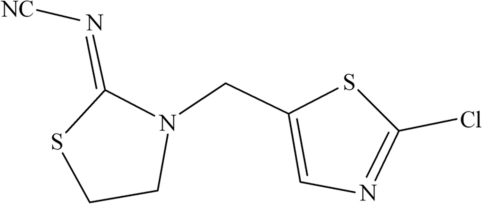


## Experimental

### 

#### Crystal data

C_8_H_7_ClN_4_S_2_
*M* *_r_* = 258.75
Monoclinic, 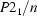

*a* = 9.6331 (12) Å
*b* = 11.2657 (14) Å
*c* = 10.7675 (13) Å
β = 112.433 (2)°
*V* = 1080.1 (2) Å^3^
*Z* = 4
Mo *K*α radiation
μ = 0.71 mm^−1^
*T* = 273 K
0.15 × 0.10 × 0.10 mm

#### Data collection

Bruker SMART CCD diffractometer
Absorption correction: multi-scan (*SADABS*; Sheldrick, 1996 ▶) *T*_min_ = 0.901, *T*_max_ = 0.932
6191 measured reflections
2440 independent reflections
2075 reflections with *I* > 2σ(*I*)
*R*_int_ = 0.015

#### Refinement

*R*[*F* ^2^ > 2σ(*F* ^2^)] = 0.033
*wR*(*F* ^2^) = 0.108
*S* = 0.98
2440 reflections
136 parameters
H-atom parameters constrained
Δρ_max_ = 0.38 e Å^−3^
Δρ_min_ = −0.27 e Å^−3^

### 

Data collection: *SMART* (Bruker, 1998 ▶); cell refinement: *SAINT* (Bruker, 1998 ▶); data reduction: *SAINT*; program(s) used to solve structure: *SHELXS97* (Sheldrick, 2008 ▶); program(s) used to refine structure: *SHELXL97* (Sheldrick, 2008 ▶); molecular graphics: *SHELXTL* (Sheldrick, 2008 ▶); software used to prepare material for publication: *SHELXTL*.

## Supplementary Material

Crystal structure: contains datablock(s) I, global. DOI: 10.1107/S1600536811047404/lh5358sup1.cif

Supplementary material file. DOI: 10.1107/S1600536811047404/lh5358Isup2.mol

Structure factors: contains datablock(s) I. DOI: 10.1107/S1600536811047404/lh5358Isup3.hkl

Supplementary material file. DOI: 10.1107/S1600536811047404/lh5358Isup4.cml

Additional supplementary materials:  crystallographic information; 3D view; checkCIF report

## Figures and Tables

**Fig. 1.**
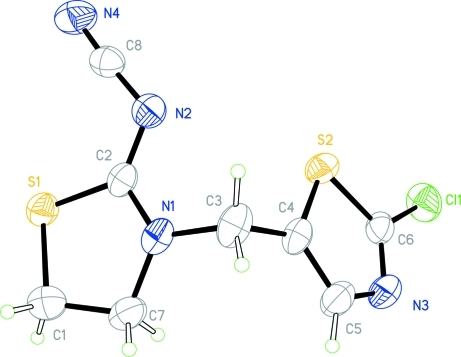
The molecular structure of the title compound showing displacement ellipsoids at the 40% probability level.

## supplementary crystallographic information

### Comment

It is already known that certain cyanoimino-subsitituted heterocyclic compounds are useful as intermediates in the preparation of pesticides which have played a major role in eliminating insects such as aphids, leafhoppers and whiteflies (Kagabu *et al.*, 2008; Zhang *et al.*, 2000). The molecular structure of the title compound is shown in Fig. 1. The thiazole ring is essentially planar (r.m.s. deviations 0.0011 (2)Å) and the thiazolidine ring is is in a slight half-chair conformation. The C═N bond with a *Z* configuration has a bond length of 1.150 (4) Å, which is in agreement with that in a related structure (Li *et al.*, 2010).

### Experimental

The synthesis of the title compound follows the method of Kozo *et al.* (1987) and Zuo, *et al.* (2008). (*Z*)-2-(1,3-Thiazolidin-2-ylidene)cyanamide 12.7 g (0.1 mol) and potassium carbonate 41.4 g (0.3 mol) were dissolved in *N*,*N*-dimethylformamide (DMF) (75 ml), then 2-chloro-5-thiazolylmethyl chloride 17.4 g (0.102 mol) dissolved in DMF (40 ml) was added dropwise. The mixture was stirred for 0.5 h at room temperature and filtered. The filtrate was concentrated and further purified by column chromatography to obtain the title product (13.9 g) with a yield of 53.7% Colorless crystals were obtained by slow evaporation of a tetrahydrofuran solution of the title compound at room temperature.

^1^H NMR (300 MHz, DMSO-d6): δ (p.p.m.) 7.71 (1*H*, s), 4.79 (2*H*, s), 3.92 (2*H*, t, *J* = 15.3 Hz, *J* = 7.65 Hz); 3.49 (2*H*, t, *J* = 15.3 Hz, *J* = 7.65 Hz).

### Refinement

All H atoms were placed in calculated positions, with C—H = 0.93-0.97 Å and included in the final cycles of refinement using a riding model, with *U*iso(H) = 1.2*U*eq(C).

### Crystal data

**Table:**

C_8_H_7_ClN_4_S_2_	*F*(000) = 528
*M**_r_* = 258.75	*D*_x_ = 1.591 Mg m^−^^3^
Monoclinic, *P*2_1_/*n*	Mo *K*α radiation, λ = 0.71073 Å
Hall symbol: -P 2yn	Cell parameters from 3349 reflections
*a* = 9.6331 (12) Å	θ = 2.7–27.5°
*b* = 11.2657 (14) Å	µ = 0.71 mm^−^^1^
*c* = 10.7675 (13) Å	*T* = 273 K
β = 112.433 (2)°	Block, colorless
*V* = 1080.1 (2) Å^3^	0.15 × 0.10 × 0.10 mm
*Z* = 4	

### Data collection

**Table:**

Bruker SMART CCD diffractometer	2440 independent reflections
Radiation source: fine-focus sealed tube	2075 reflections with *I* > 2σ(*I*)
graphite	*R*_int_ = 0.015
φ and ω scans	θ_max_ = 27.5°, θ_min_ = 2.4°
Absorption correction: multi-scan (*SADABS*; Sheldrick, 1996)	*h* = −12→12
*T*_min_ = 0.901, *T*_max_ = 0.932	*k* = −11→14
6191 measured reflections	*l* = −13→13

### Refinement

**Table:**

Refinement on *F*^2^	Primary atom site location: structure-invariant direct methods
Least-squares matrix: full	Secondary atom site location: difference Fourier map
*R*[*F*^2^ > 2σ(*F*^2^)] = 0.033	Hydrogen site location: inferred from neighbouring sites
*wR*(*F*^2^) = 0.108	H-atom parameters constrained
*S* = 0.98	*w* = 1/[σ^2^(*F*_o_^2^) + (0.0632*P*)^2^ + 0.5077*P*] where *P* = (*F*_o_^2^ + 2*F*_c_^2^)/3
2440 reflections	(Δ/σ)_max_ < 0.001
136 parameters	Δρ_max_ = 0.38 e Å^−^^3^
0 restraints	Δρ_min_ = −0.27 e Å^−^^3^

### Special details

**Table:**

Geometry. All e.s.d.'s (except the e.s.d. in the dihedral angle between two l.s. planes) are estimated using the full covariance matrix. The cell e.s.d.'s are taken into account individually in the estimation of e.s.d.'s in distances, angles and torsion angles; correlations between e.s.d.'s in cell parameters are only used when they are defined by crystal symmetry. An approximate (isotropic) treatment of cell e.s.d.'s is used for estimating e.s.d.'s involving l.s. planes.
Refinement. Refinement of *F*^2^ against ALL reflections. The weighted *R*-factor *wR* and goodness of fit *S* are based on *F*^2^, conventional *R*-factors *R* are based on *F*, with *F* set to zero for negative *F*^2^. The threshold expression of *F*^2^ > σ(*F*^2^) is used only for calculating *R*-factors(gt) *etc*. and is not relevant to the choice of reflections for refinement. *R*-factors based on *F*^2^ are statistically about twice as large as those based on *F*, and *R*- factors based on ALL data will be even larger.

### Fractional atomic coordinates and isotropic or equivalent isotropic displacement parameters (Å^2^)

**Table:**

	*x*	*y*	*z*	*U*_iso_*/*U*_eq_	
S1	0.22859 (6)	0.11411 (5)	0.45559 (5)	0.04850 (16)	
S2	0.42112 (6)	0.16077 (5)	0.07049 (5)	0.04881 (17)	
Cl1	0.30959 (8)	0.27500 (7)	−0.19805 (6)	0.0715 (2)	
N1	0.3166 (2)	−0.00659 (14)	0.29672 (16)	0.0445 (4)	
C4	0.3566 (2)	0.02118 (18)	0.08619 (19)	0.0452 (4)	
C2	0.3576 (2)	0.08458 (16)	0.38105 (18)	0.0398 (4)	
C3	0.4033 (3)	−0.0430 (2)	0.2174 (2)	0.0532 (5)	
H3A	0.3906	−0.1277	0.2004	0.064*	
H3B	0.5090	−0.0282	0.2688	0.064*	
C1	0.1211 (3)	−0.0170 (2)	0.3809 (3)	0.0680 (7)	
H1A	0.1433	−0.0804	0.4466	0.082*	
H1B	0.0144	0.0000	0.3481	0.082*	
N2	0.4818 (2)	0.14397 (16)	0.40261 (18)	0.0485 (4)	
C6	0.3112 (2)	0.1563 (2)	−0.09762 (19)	0.0475 (5)	
N3	0.2328 (2)	0.0626 (2)	−0.14132 (17)	0.0598 (5)	
C8	0.5191 (2)	0.22657 (19)	0.4975 (2)	0.0484 (5)	
N4	0.5597 (3)	0.29942 (19)	0.5786 (2)	0.0680 (6)	
C7	0.1657 (3)	−0.05279 (19)	0.2663 (2)	0.0527 (5)	
H7A	0.1649	−0.1385	0.2579	0.063*	
H7B	0.0961	−0.0197	0.1824	0.063*	
C5	0.2592 (3)	−0.0145 (2)	−0.0353 (2)	0.0580 (6)	
H5A	0.2118	−0.0880	−0.0471	0.070*	

### Atomic displacement parameters (Å^2^)

**Table:**

	*U*^11^	*U*^22^	*U*^33^	*U*^12^	*U*^13^	*U*^23^
S1	0.0521 (3)	0.0483 (3)	0.0467 (3)	0.0000 (2)	0.0207 (2)	−0.0068 (2)
S2	0.0445 (3)	0.0554 (3)	0.0384 (3)	−0.0010 (2)	0.0067 (2)	−0.0027 (2)
Cl1	0.0720 (4)	0.0877 (5)	0.0533 (3)	0.0002 (3)	0.0224 (3)	0.0209 (3)
N1	0.0568 (10)	0.0397 (8)	0.0339 (7)	0.0054 (7)	0.0139 (7)	−0.0003 (6)
C4	0.0520 (11)	0.0470 (10)	0.0371 (9)	0.0097 (8)	0.0176 (8)	−0.0041 (8)
C2	0.0474 (10)	0.0364 (9)	0.0315 (8)	0.0081 (7)	0.0104 (7)	0.0059 (7)
C3	0.0686 (14)	0.0465 (11)	0.0433 (10)	0.0187 (10)	0.0200 (10)	0.0000 (9)
C1	0.0731 (16)	0.0668 (15)	0.0673 (15)	−0.0213 (13)	0.0304 (13)	−0.0164 (12)
N2	0.0487 (9)	0.0484 (9)	0.0476 (9)	0.0013 (7)	0.0174 (8)	0.0004 (8)
C6	0.0418 (10)	0.0644 (13)	0.0353 (9)	0.0028 (9)	0.0137 (8)	0.0014 (9)
N3	0.0615 (11)	0.0772 (13)	0.0347 (8)	−0.0110 (10)	0.0114 (8)	−0.0067 (9)
C8	0.0432 (10)	0.0453 (11)	0.0526 (11)	−0.0015 (8)	0.0139 (9)	0.0059 (9)
N4	0.0686 (13)	0.0557 (12)	0.0721 (13)	−0.0149 (10)	0.0185 (11)	−0.0115 (10)
C7	0.0626 (13)	0.0431 (10)	0.0436 (10)	−0.0065 (9)	0.0105 (9)	−0.0029 (9)
C5	0.0721 (14)	0.0561 (13)	0.0446 (11)	−0.0100 (11)	0.0210 (10)	−0.0122 (10)

### Geometric parameters (Å, °)

**Table:**

S1—C2	1.749 (2)	C3—H3B	0.9700
S1—C1	1.807 (2)	C1—C7	1.508 (3)
S2—C6	1.714 (2)	C1—H1A	0.9700
S2—C4	1.723 (2)	C1—H1B	0.9700
Cl1—C6	1.716 (2)	N2—C8	1.326 (3)
N1—C2	1.327 (2)	C6—N3	1.279 (3)
N1—C7	1.459 (3)	N3—C5	1.378 (3)
N1—C3	1.463 (3)	C8—N4	1.153 (3)
C4—C5	1.348 (3)	C7—H7A	0.9700
C4—C3	1.495 (3)	C7—H7B	0.9700
C2—N2	1.313 (3)	C5—H5A	0.9300
C3—H3A	0.9700		
			
C2—S1—C1	91.54 (11)	S1—C1—H1A	110.4
C6—S2—C4	88.65 (10)	C7—C1—H1B	110.4
C2—N1—C7	116.13 (17)	S1—C1—H1B	110.4
C2—N1—C3	121.99 (18)	H1A—C1—H1B	108.6
C7—N1—C3	120.70 (17)	C2—N2—C8	117.03 (18)
C5—C4—C3	128.3 (2)	N3—C6—S2	116.92 (17)
C5—C4—S2	108.70 (17)	N3—C6—Cl1	123.34 (16)
C3—C4—S2	123.00 (16)	S2—C6—Cl1	119.75 (13)
N2—C2—N1	121.90 (18)	C6—N3—C5	108.62 (18)
N2—C2—S1	125.56 (15)	N4—C8—N2	175.7 (2)
N1—C2—S1	112.54 (15)	N1—C7—C1	106.93 (17)
N1—C3—C4	112.53 (17)	N1—C7—H7A	110.3
N1—C3—H3A	109.1	C1—C7—H7A	110.3
C4—C3—H3A	109.1	N1—C7—H7B	110.3
N1—C3—H3B	109.1	C1—C7—H7B	110.3
C4—C3—H3B	109.1	H7A—C7—H7B	108.6
H3A—C3—H3B	107.8	C4—C5—N3	117.1 (2)
C7—C1—S1	106.81 (17)	C4—C5—H5A	121.4
C7—C1—H1A	110.4	N3—C5—H5A	121.4
			
C6—S2—C4—C5	−0.26 (17)	N1—C2—N2—C8	−174.14 (18)
C6—S2—C4—C3	−178.81 (18)	S1—C2—N2—C8	6.2 (3)
C7—N1—C2—N2	−170.80 (17)	C4—S2—C6—N3	0.15 (19)
C3—N1—C2—N2	−3.1 (3)	C4—S2—C6—Cl1	179.78 (14)
C7—N1—C2—S1	8.9 (2)	S2—C6—N3—C5	0.0 (3)
C3—N1—C2—S1	176.56 (14)	Cl1—C6—N3—C5	−179.61 (17)
C1—S1—C2—N2	−174.02 (19)	C2—N1—C7—C1	−22.8 (2)
C1—S1—C2—N1	6.31 (16)	C3—N1—C7—C1	169.36 (19)
C2—N1—C3—C4	−88.0 (2)	S1—C1—C7—N1	25.2 (2)
C7—N1—C3—C4	79.1 (2)	C3—C4—C5—N3	178.8 (2)
C5—C4—C3—N1	−95.7 (3)	S2—C4—C5—N3	0.3 (3)
S2—C4—C3—N1	82.6 (2)	C6—N3—C5—C4	−0.2 (3)
C2—S1—C1—C7	−18.40 (19)		

## References

[bb1] Bruker (1998). *SMART* and *SAINT*, Bruker AXS Inc., Madison, Wisconsin, USA.

[bb2] Kagabu, S., Nishimura, K., Naruse, Y. & Ohno, I. (2008). *J. Pestic. Sci* **33**, 58–66.

[bb3] Kozo, S., Shinichi, T., Shinzo, K., Shoko, S., Koichi, M. & Yumi, H. (1987). EP Patent 235725.

[bb4] Li, H., Zhang, X. & Xu, L. (2010). *Acta Cryst.* E**66**, o2171. 10.1107/S1600536810029879 PMC3007229 21588452

[bb5] Sheldrick, G. M. (1996). *SADABS* University of Göttingen, Germany.

[bb6] Sheldrick, G. M. (2008). *Acta Cryst.* A**64**, 112–122. 10.1107/S0108767307043930 18156677

[bb7] Zhang, A. G., Kayser, H., Maiensch, P. & Casida, J. E. (2000). *J. Neurochem.* **75**, 1294–1303. 10.1046/j.1471-4159.2000.751294.x 10936213

[bb8] Zuo, B. J., Shi, L. P., Li, L., Li, X. K. & Zhuang, Z. X. (2008). CN Patent 101250165.

